# Use of the Growth Hormone Stimulation Test Result in the Management of Patients With a Short Stature

**DOI:** 10.7759/cureus.10988

**Published:** 2020-10-16

**Authors:** Moeber M Mahzari, Futun Al Joufi, Shams Al Otaibi, Esra Hassan, Emad Masuadi

**Affiliations:** 1 Medicine, College of Medicine, King Saud Bin Abdulaziz University for Health Sciences, Riyadh, SAU; 2 Medicine, Ministry of National Guard - Health Affairs, Riyadh, SAU; 3 Medicine, King Abdullah International Medical Research Center, Riyadh, SAU; 4 Pediatrics, Ministry of National Guard - Health Affairs, Riyadh, SAU; 5 Pediatrics, King Abdullah International Medical Research Center, Riyadh, SAU; 6 Otolaryngology, King Fahad Medical City, Riyadh, SAU; 7 Biostatistics, King Saud Bin Abdulaziz University for Health Sciences, Riyadh, SAU

**Keywords:** growth hormone, growth hormone stimulation test, short stature (ss)

## Abstract

Introduction

A proportionate short stature (SS) assessment involves the documentation of normal growth hormone secretion via a growth hormone (GH) stimulation test. All available GH stimulation tests have some disadvantages. The decision to initiate GH therapy is dependent on multiple factors, including the GH stimulation test result. However, many patients receive GH therapy, even if they have a normal GH stimulation test result, with the indication of a presumed idiopathic SS.

Objective

In this study, we investigated the use of the GH stimulation test result in initiating GH therapy.

Method

A cross-sectional study was conducted with patients diagnosed with proportionate SS. Age, gender, insulin-like growth factor 1 (IGF-1) level, and GH stimulation test results were collected retrospectively from the electronic medical records. The main outcome variable was the decision related to prescribing GH therapy.

Results

A total of 286 patient charts were reviewed, and the majority (n = 201, 64.6%) were male. For just less than half (n = 136, 47.6%), the result of the GH stimulation test was ≥ 10 ng/mL, in a small proportion (n = 53, 18.5%) the result was < 5 ng/mL, and for the rest of the cohort, the result was 5.0 - 9.9 ng/mL. The majority (n = 219, 70.4%) received GH therapy, irrespective of the GH stimulation test result. The odds ratio (OR) for GH treatment was 3.9 (CI: 1.79 - 8.49) and 3.0 (CI: 1.21 - 7.42) for patients with a result < 5 ng/mL and 5.0 - 9.9 ng/mL, respectively, compared to the group with a result of ≥ 10 ng/mL.

Conclusion

GH therapy is frequently prescribed for patients with SS, irrespective of the GH stimulation test result. However, the group with SS with a result of < 9.9 ng/mL was more likely to receive GH therapy. The question of whether a GH stimulation test is required, in the context of SS, is debatable.

## Introduction

A short stature (SS) is a frequent concern presented to pediatricians and endocrinologists [1]. SS is defined as height less than two standard deviations below the median appropriate height for a population, taking into consideration the patient’s gender and age. SS could also be defined as a height below the 3rd percentile on a growth chart, or a growth velocity below the 25th percentile consistently over six to 12 months of observation [1-2]. The global prevalence of SS is 3% [3]. The SS prevalence in Saudi children and adolescents is considered to be similar to the global prevalence, but the exact prevalence is not known [4]. SS is classified as disproportionate and proportionate SS. Disproportionate SS, a rare type, presents with short limbs, such as diastrophic dysplasia and chondrodysplasia punctata, or a short trunk, such as in mucopolysaccharidosis. The more prevalent normal variant SS is classified as the proportionate type, with normal proportionate growth of limbs and trunk. The causes of proportionate SS include prenatal causes (genetic disorders) and postnatal causes, such as growth hormone deficiency (GHD). GHD classically could be deficient in isolation or with other pituitary hormones [3].

The diagnosis of GHD is challenging due to the pulsatile nature of growth hormone secretion in the body. It is also dependent on age, gender, and body mass index (BMI). A single random measurement of growth hormone is rarely diagnostic. Biochemical growth hormone markers, such as the insulin-like growth factor 1 (IGF-1) and the IGF-binding protein 3 (IGFBP-3), have poor diagnostic values due to overlapping results of the markers in healthy and GHD patients. According to the Growth Hormone Research Society, one dependable growth hormone stimulation test is adequate to make the diagnosis. In the United States, an insulin tolerance test (ITT) is the gold standard stimulation test for adults with GHD [5]. The cut-off point for the diagnosis is a GH level of 3 to 5 ng/mL during acute hypoglycemia, i.e., less than 40 mg/dL. However, ITT is contraindicated in coronary artery disease and epileptic patients. A major drawback of this test is that it requires close monitoring due to adverse effects, such as seizures and loss of consciousness. The glucagon stimulation test (GST) is an acceptable alternative test to ITT. GST is more potent in stimulating GH in comparison to other stimulation tests, such as clonidine. In addition, the intramuscular or subcutaneous route of administration of GST is more effective than the intravenous route. Reproducibility and safety are the main advantages of GST. The relatively long duration of the procedure, up to four hours, is considered a disadvantage of the test [5]. The clonidine stimulation test (CST) is the preferred test in diagnosing children with GHD [6]. Clinically, there is no standardized peak time with CST as the GH response peak time could be early or late. CST involves an oral dose of 150 μg/m^2^ of clonidine and the GH response is measured at successive periods of 0, 30, 45, 60, and 90 minutes. Measuring the GH response at different times can be financially demanding. Thakur et al. found that the 60 minutes post-CST measurement is the most important to exclude GHD (79.5% specificity). The addition of the 90 minutes raises the test’s specificity to 92.3% [6].

IGFs are GH-dependent peptide factors that mediate the actions of GH [7]. As IGFs levels remain steady during the day, it can be assessed with a single estimation [8]. The pulsatile section rhythm of GH and the fact that it is low most of the day mandates the stimulation test of GH [9]. However, IGF-1 in the pediatric age group lacks the specificity as a reduced level could occur in other endocrine and nutritional abnormalities [10-12]. In addition, it is significantly affected by the age and pubertal status of the patient as it could overlap with normality [13-15]. A number of studies concluded that IGF-1 in pediatric age doses does not correlate with the GH status [16-18]. In practice, more information related to the case is collected from a single IGF-1 and IGFBP-3 measurement, in combination with a single GH stimulation test. For a subnormal GH stimulation test result, a second stimulation test supports the differentiation between GHD and a normal SS [9]. The treatment for patients with SS due to GHD is GH injections. It should be noted that during the last decade, GH treatment for SS expanded and is indicated for almost all cases of proportionate SS, not only in patients with GHD [1]. Currently, patients with SS with a normal stimulation test (GHD not diagnosed based on the GH stimulation test) are candidates for GH treatment for presumed idiopathic SS. This change in GH indications over time may render the GH stimulation test redundant in the context of SS as most, if not all, the patients with proven proportionate SS are candidates to receive GH therapy. In this study, we assessed the impact of the GH stimulation test result on the decision to treat with GH in a relatively large group of patients with a diagnosis of SS.

## Materials and methods

A cross-sectional study was conducted using a retrospective chart review of patients attending endocrine clinics with a diagnosis of SS. The study was conducted at King Abdulaziz Medical City in Riyadh, Saudi Arabia. King Abdulaziz Medical City is one of the largest medical cities in Saudi Arabia with primary, secondary, and tertiary care facilities.

All patients, four to 18 years old, with a confirmed diagnosis of SS by an endocrinologist and who had a GH stimulation test of any type from 2000 to 2016 were included in the study. Patients with a comorbid condition, such as chronic kidney disease, as a cause of SS, were excluded from the study. The data collected included the age of diagnosis with SS, gender, type of GH stimulation test used, the GH response to the stimulation test, the IGF-1 level at diagnosis, and whether the patient received GH therapy or not. The main outcome variable was the categorical variable of treatment with GH or not. The data were collected with a structured data collection sheet and analyzed by the Statistical Package for Social Sciences (SPSS), v. 20 (IBM SPSS Statistics, Armonk, NY). Percentage and frequency were used to describe categorical variables and a mean and standard deviation for the numerical variables. A Chi-square test was used to compare the significance of the outcome variable between different groups. A statistical test with a p-value of less than 0.05 was considered statistically significant.

## Results

A total of 286 patient charts were reviewed. The majority of the patients (n = 201, 64.6%) were male and 175 patients (56.3%) were ≥ 10 years old (Table 1).

**Table 1:** Demographical Data of the Patients GH: growth hormone; IGF-1: insulin-like growth factor 1; ITT: insulin tolerance test; SD: standard deviation

Variable	Categories	N	%
Gender	Male	201	64.6
	Female	110	35.4
Age (years)	< 10	136	43.7
	≥ 10	175	56.3
Type of GH stimulation test	Clonidine	205	65.9
	Glucagon	88	28.3
	ITT	18	5.8
Peak GH after stimulation (ng/mL)	10+	136	47.6
	5 - 9.9	97	33.9
	< 5	53	18.5
Pt given GH	Yes	219	70.4
	No	92	29.6
IGF-1 level (ng/mL) (Mean ± SD	107.45 ± 72.2		

The most frequently used GH stimulation test was the clonidine stimulation test (n = 205, 65.9%), followed by the GST (n = 88, 28.3%), and only a small proportion (n = 18, 5.8%) of the sample had an ITT. For less than half of the patients (n = 136, 47.6%), the GH stimulation test result was ≥ 10 ng/mL. In a small proportion (n = 53, 18.5%), the result was < 5 ng/mL. For the rest of the cohort, the result was between 5.0 - 9.9 ng/mL.

Table 2 displays the proportions of the patients who received GH treatment, grouped by their GH stimulation test result. The majority of the sample (n = 219, 70.4%) received GH therapy. For the ≥ 10 ng/mL stimulation group (n = 136), more than half (n = 79, 58.1%) received GH treatment; in the < 5 ng/mL stimulation group (n = 53), 46 (86.6%) patients received GH. A similar pattern was also seen in the 5.0 - 9.9 ng/mL group (n = 97), with 84 (86.6%) receiving GH. The outcome variable (GH therapy prescribed or not) assessed with a Chi-square test was statistically different between the groups (p-value of < 0.5).

**Table 2:** The Association Between the Peak Growth Hormone (GH) After Stimulation and Whether GH Was Given or Not

	Patient given GH				P-value
	Yes		No		
Peak GH after stimulation (ng/mL)	N	%	N	%	
10+	79	58.1	57	41.9	< 0.001
5 - 9.9	84	86.6	13	13.4	
< 5	46	86.8	7	13.2	
Total	209	73.1	77	26.9	

The mean level of IGF-1 at the time of diagnosis was not significantly different between the groups who received or did not receive GH (108 versus 105 ng/mL).

The only factor that significantly affecting the GH therapy, was a GH stimulation result 9.9 ng/mL or lower. The odds ratio (OR) to receive GH treatment in patients with a result < 5 ng/mL was 3.9 (CI: 1.79 - 8.49) when the stimulated GH ≥ 10 ng/mL group was used as the reference group in the logistic regression analysis. The same pattern was observed with an OR of 3.0 (CI: 1.21 - 7.42) for the 5.0 - 9.9 ng/mL group, when referenced to the ≥ 10 ng/mL group. Age, gender, and IGF-1 level at the time of the diagnosis did not influence the GH therapy significantly (Table 3).

**Table 3:** Logistic Regression of Gender, Age, IGF-1 Level, and Growth Hormone (GH) Stimulation Result Dependent variable is the status of growth hormone therapy (whether GH was given or not) * is the reference group CI: confidence interval; IGF-1: insulin-like growth factor 1; OR: odds ratio

		P-value	OR	95% CI for OR	
				Lower	Upper
Gender	Male	0.432	0.75	0.37	1.54
	Female*		1		
Peak GH after stimulation (ng/mL)	< 5	0.001	3.9	1.79	8.49
	5 - 9.9	0.018	3	1.21	7.42
	10+*		1		
Age (years)	< 10	0.628	1.19	0.60	2.36
	≥ 10*		1		
IGF-1 level at diagnosis		0.51	1.0	0.99	1.00

## Discussion

The decision to treat SS patients with GH is, to a large extent, subjective and is based on multiple variables. One of these variables is the result of the GH stimulation test, which is indicated in all patients with proportionate SS. It is noteworthy that GH treatment is justified in all patients with SS if there are no contraindications. Even patients with a normal growth hormone stimulation are candidates for GH treatment for presumed idiopathic SS [1]. The objective of this study was to assess the impact of the GH stimulation test results on the use of GH therapy in a large cohort of patients with SS at a tertiary care center, considering other available variables.

The study results indicated that the majority of the patients in this study received GH treatment, regardless of the GH stimulation test result, supporting the notion that GH therapy is an option for all SS patients if there are no contraindications. However, there was a significant difference between the groups with a test result < 10 ng/mL as more of this group received GH therapy compared to the group with a result ≥ 10 ng/mL. The difference was expected. However, if we focus on the group with a result ≥ 10 ng/mL, the majority also received GH therapy despite the normal stimulated GH test result. A small proportion (13%) of the patients with a subnormal GH response did not receive GH therapy, indicating that the GH stimulation test result is not the only or the most important factor influencing the GH therapy decision.

GH treatment should be initiated as early as possible to maximize the benefit of GH and to promote the normalization of the stature prior to adulthood, taking into consideration the child’s genetic background [19-20]. If the logistics required for a GH stimulation test, the possible risks, and the delay of the initiation of GH therapy due to the GH stimulation test are considered, we submit that the GH stimulation test could be omitted in most patients with SS. Indeed, in countries in the Middle East where the GH stimulation test is not readily available, patients may have to wait many months for the test before initiating GH therapy. This delay, especially in patients who are approaching complete bone maturity, might be harmful. The timely initiation of GH therapy without a GH stimulation test is justified due to the practice to initiate GH therapy regardless of the test result.

The importance of the GH stimulation test in the context of SS is to identify patients with GHD who will require longer GH therapy, even after height completion. However, the dependence on the GH stimulation test as the only way to confirm or diagnose GHD is debatable. The use of GH replacement after growth completion in patients with possible isolated GHD as children or adolescents is not recommended unless the GHD is still present after height completion. In patients with an isolated GH deficiency, the GH stimulation test should be repeated at approximately 18 years to guide the decision to continue with GH replacement, highlighting the minor impact of the initial GH stimulation test on the long-term continuation of GH therapy [21]. Another important argument is that the GH stimulation test result may guide the GH dose, but this application is also questionable as there is an overlap in the GH dose required in specific conditions. The recommendation is to dose GH as weight-based or body surface area (BSA)-based, and it is, to a large extent, physician and institution-dependent [22-26]. The IGF-1 can serve as a tracking biomarker for the GH therapy, to evaluate its effectiveness, monitor adherence, and titrate the dose as appropriate [20, 26].

Though the study included a large sample from a single tertiary center, we acknowledge the limitations of conducting a retrospective study, which may lack important data required for a robust conclusion. In particular, accurate data on the patients’ height, growth velocity, and height change over time, in relation to the GH stimulation test results and their effect on the GH therapy decision, were not available. Additional prospective studies, focusing on the impact of the GH stimulation test on the SS treatment, particularly in patients with a normal GH stimulation result, are required. It is essential to determine the utility of the GH stimulation test in relation to the initiation and guidance of GH therapy and the disadvantage of delaying the timely initiation of GH therapy.

## Conclusions

GH therapy is frequently prescribed for patients with SS, irrespective of the GH stimulation test result. However, the group with SS with a result of < 9.9 ng/mL was more likely to receive GH therapy. The question of whether a GH stimulation test is required, in the context of SS, is debatable.
